# Comparison of Polymeric Cyanoacrylate Adhesives with Suturing in Free Gingival Graft Stability: A Split Mouth Trial

**DOI:** 10.3390/polym13203575

**Published:** 2021-10-16

**Authors:** Reham N. AlJasser, Mohammed A. AlSarhan, Dalal H. AlOtaibi, Saleh AlOraini, Rand AlNuwaiser, Alanoud AlOtaibi, Hessah Alduraihem, Syed Rashid Habib, Muhammad Sohail Zafar

**Affiliations:** 1 Department of Periodontics and Community Dentistry, College of Dentistry, King Saud University, Riyadh 11545, Saudi Arabia; raljasser@ksu.edu.sa (R.N.A.); malsarhan@ksu.edu.sa (M.A.A.); dalalotaibi@ksu.edu.sa (D.H.A.); ssaloraini@ksu.edu.sa (S.A.); 2 Undergraduate Dental Student, College of Dentistry, King Saud University, Riyadh 11545, Saudi Arabia; 438202871@student.ksu.edu.sa (R.A.); 438201444@student.ksu.edu.sa (A.A.); 438201523@student.ksu.edu.sa (H.A.); 3 Department of Prosthetic Dental Sciences, College of Dentistry, King Saud University, Riyadh 11545, Saudi Arabia; 4 Department of Restorative Dentistry, College of Dentistry, Taibah University, Al Madinah, Al Munawwarah 41311, Saudi Arabia; MZAFAR@taibahu.edu.sa; 5 Department of Dental Materials, Islamic International Dental College, Riphah International University, Islamabad 44000, Pakistan

**Keywords:** cyanoacrylate, cyanoacrylate adhesive, free gingival grafts, suturing techniques, wound healing, gingival grafts

## Abstract

The aim is to compare the use of Cyanoacrylate adhesives (CAA) to the conventional suturing technique in terms of free gingival grafts (FGG) stability and healing in lower anterior and premolar regions. A split mouth design was initiated on 22 participants. Each side (from 2nd premolar to central incisor) was randomized to either the control or test groups. In the control group, sutures were used to stabilize the FGG, while, in the test group, the FGG was stabilized with butyl-cyanoacrylate. Full-periodontal clinical parameters were employed to assess the periodontal health. FGG-related parameters assessed included the keratinized tissue width (KTW), gingival tissue thickness (GTT), FGG shrinkage% and pain using the VAS score. No significant differences in the mean values of the KTW nor FGG shrinkage% across six time points (*p* < 0.05) were observed, whereas highly significant differences in the mean values of GTT across six time points (F = 3.32; *p* = 0.008) were observed. The use of CAA in FGG stability and healing is comparable to conventional suturing for soft tissue grafts in terms of success outcomes. With its cost effectiveness, lesser time consumption, post-operative pain and comparable graft stability and dimensions, the use of CAA may be a promising alternative for conventional and microsurgical techniques for the stabilization of FGG in the oral cavity.

## 1. Introduction

A free gingival graft (FGG) is a periodontal surgical procedure utilized to create vestibular depth and widen the zone of the keratinized tissue, and was one of the first procedures used to treat gingival recession as well [1,2]. The importance of this procedure is to mainly increase the amount of attached tissue, which in turn increases the patient’s ability to clean and remove plaque, leading to a reduction in gingival inflammation and, in addition, to improve aesthetics [2]. As any other surgical periodontal procedure, the FGG has some challenges such as the need for a second surgical area (donor site) and the necessity of proper suturing, which may cause some inconvenience to the patient and sloughing to the graft due to vasoconstriction and the loss of a proper blood supply to the graft, especially when applied under tension [3].

Developing successful and satisfactory outcomes with an FGG depends on several factors, including harvested tissue dimensions (width, height and thickness), a proper preparation of the recipient site and, mostly, the stability of the graft when placed with no movement observed [4]. Graft stability and minimal movement are crucial to maintain an adequate vascularity during the healing period, which, in turn, ensure the success of the procedure. Another important factor to consider is to not strangulate the graft by over suturing or stretching [5]. Therefore, having an equalized distribution of pressure points along the graft is essential and must be considered by the periodontist [6].

Several suturing techniques have been proposed to optimize the stability and vascularity needed to be obtained, such as the continuous horizontal suture proposed by Holbrook and Ochsenbein [7], and the apical stretching suture proposed by Miller [8]. In addition, other modalities were further investigated, which included the use of Cyanoacrylate adhesives (CAA) to stabilize the graft instead of sutures [9,10,11]. CAA is a material composed of synthesized monomers, cyanoacetate and formaldehyde mixed in the existence of catalysts to form an adhesive film that forms by quick polymerization and is aggravated by hydroxyl groups to be glued [9]. The chemical formula is CH2=C(CN)-COOR, where R donates any alkyl group, ranging from methyl to decyl. Altering the type of alkyl chains with a longer molecular chain may reduce the tissue toxicity [10,11,12,13]. N-butyl cyanoacrylate was comprehensively tested and found to be biocompatible in human tissues and exhibited to be effective as an adhesive with bacteriostatic and hemostatic properties. Compared to conventional suture materials used in soft-tissue surgery, which can be an additional superior factor to a conventional suturing material, other advantages included time efficiency due to the easiness of use and the prompt setting occurring after its application and, finally, the lack of the need to be removed during a post-operative follow-up. On the other hand, several side effects were reported, such as irreversible retinal damage having occurred; therefore, patients are instructed to wear eye protection during the procedure [13,14,15]. Furthermore, CAA is a biomaterial that can be a possible alternative to conventional sutures to stimulate a better blood supply at the grafted site and, therefore, superior healing, adding more efficiency and experiencing fewer complications [9,10,11,12,13,14,15,16,17].

Another development in the suture-less technique is the use of the Fibrin Fibronectin Sealing System (FFSS) [18]. A fibrin sealant is a synthetic material used for creating a fibrin clot. It is a combination of fibrinogen plus thrombin, in which the thrombin acts as an enzyme and converts the fibrinogen to fibrin, which can act as a tissue adhesive. A fibrin sealant in addition to an adhesive property also possesses an anti-enzymatic effect, which promotes fibroblast aggregation, their growth and adhesion [19,20].

Due to the scarcity of literature regarding the use of CAA for wound closure/healing, in the FGG used in dentistry and the lack of information on the benefits and use of CAA as compared to conventional sutures, further experimentation is needed. Therefore, the aim of the present study is to compare the use of CAA to the conventional suturing technique in terms of FGG stability and healing outcomes when applied in lower anterior and premolar areas with a reduced keratinized tissue width.

## 2. Materials and Methods

### 2.1. Study Design and Participants

The project was granted approval by the institutional review board (IRB) and its project activities were approved by the institutional committee of research ethics at the College of dentistry research center, King Saud University, Riyadh, Saudi Arabia (Reg. # E-64-8791). The study was conducted in accordance with the Helsinki Declaration of 1975, as revised in 2013. Participation in the study was completely voluntary. Individuals who sought treatment at the department of periodontology clinics located at the college of dentistry in King Saud University, Riyadh, Saudi Arabia, and showed interest in the study, were assessed against an inclusion and exclusion criterion (Table 1). Those who fulfilled all the requirements were selected to participate in the trial and signed relative informed consent documents prior to interventions. Communication was performed for each individual to cover all information in regard to the proposed surgical procedures and related clinical parameters, study objectives, design, risks and potential benefits.

A split mouth design was initiated on 22 participants. The sample size of 22 patients was calculated based on effect size of 0.5, power of 0.80 and α = 0.05 using G-Power power analysis software (G*Power 3.1.9.7, Franz Faul, Universitat Kiel, Keil, Germany). Each side (from 2nd premolar to central incisor) was randomized to either the control or test group via a randomization table. The treatment codes (CAA test/sutures control) were available in closed envelopes which were sealed. A blinded dental assistant opened it before the surgery [14].

### 2.2. Surgical Protocol for FGG

All interventions were performed under the administration of local anesthesia (Xylocaine, 2% injection with 1:100,000 epinephrine, Dentsply Pharmaceutical, York, PA, USA) (36 mg Xylocaine per carpule) as buccal and lingual infiltration in recipient site, followed by palatal infiltration in donor site with strict clinical infection control protocol. Both test and control sites were treated in the same dental appointment by the same periodontist (R.A).

**Figure 1:**
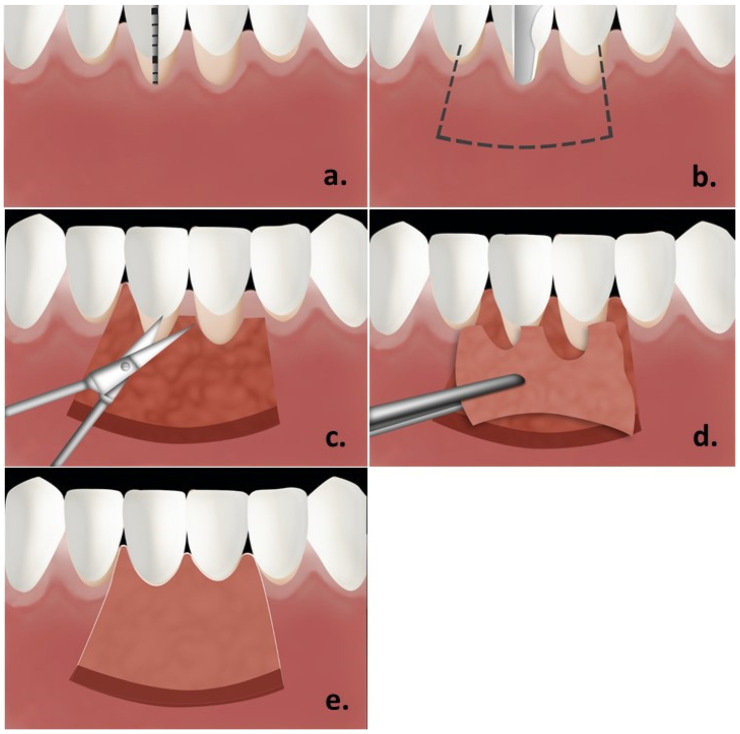
Steps of surgical procedure: (**a**).—determining the area for graft/; (**b**).—partial thickness flap design/; (**c**).—soft tissue tags removed/; (**d**,**e**).—free gingival graft in place.

FGG procedures were performed according to surgical technique proposed by Sullivan and Atkins, 1968 [1]. In regard to preparation of recipient site, prior to making the first incision, tension was applied to mucosa on mucogingival line by retracting the lip or cheek. All cases were indicated for FGG in lower anterior teeth # 35-45. First incision was performed while the tissue was still being retracted using no. 15C scalpel blade (Dentsply, York, PA, USA). The first step was to determine actual graft size needed using William’s periodontal probe (Hu-Friedy Mfg. Co., Chicago, IL 60618-5935, USA). First incision was initiated at the distal end of the surgical site having the blade held parallel to alveolar process. Full thickness incision was performed at mucogingival junction. The mucosal tissue was immediately moved apically, separated and retracted. The blade continued to be moved in a mesial direction to achieve desired length of the incision. Once the incision was completed, sharp dissection was continued apically to separate remaining alveolar mucosa from underlying periosteum. Then, periosteal bed was created in an occluso-apical direction for 5 to 8 mm. Residual soft tissue tags were removed from periosteal bed and final blending of tissue was done. Suturing the edges of the mucosal flap apically was performed using 4-0 vicryl sutures (Ethicon, Johnson & Johnson, New Brunswick, NJ, USA) as a continuous suture. As for donor site, bleeding points were introduced on palatal tissue to locate the actual graft’s size. An incision was then begun along in an occlusal direction toward the palate with a no.15 scalpel blade (Dentsply, USA) held nearly parallel to the tissue and 3 mm away from gingival margins of first and second molars. Once the first incision was completed, the blade was moved apically, causing detachment of the graft as it moved toward the apical direction. The most anterior vertical incision was conducted prior to detaching the graft apically. Then, tissue tweezer was used to hold the graft distally as it was being separated apically and dissected, until the graft was totally freed. FGG was then placed on a moistened gauze with saline. Sharp edges of FGG were trimmed and thickness was also confirmed to ensure that it was generally smooth and uniform. Final thickness reached was 1 mm (Figure 1).

### 2.3. Test vs. Control Groups Interventions

**Figure 2:**
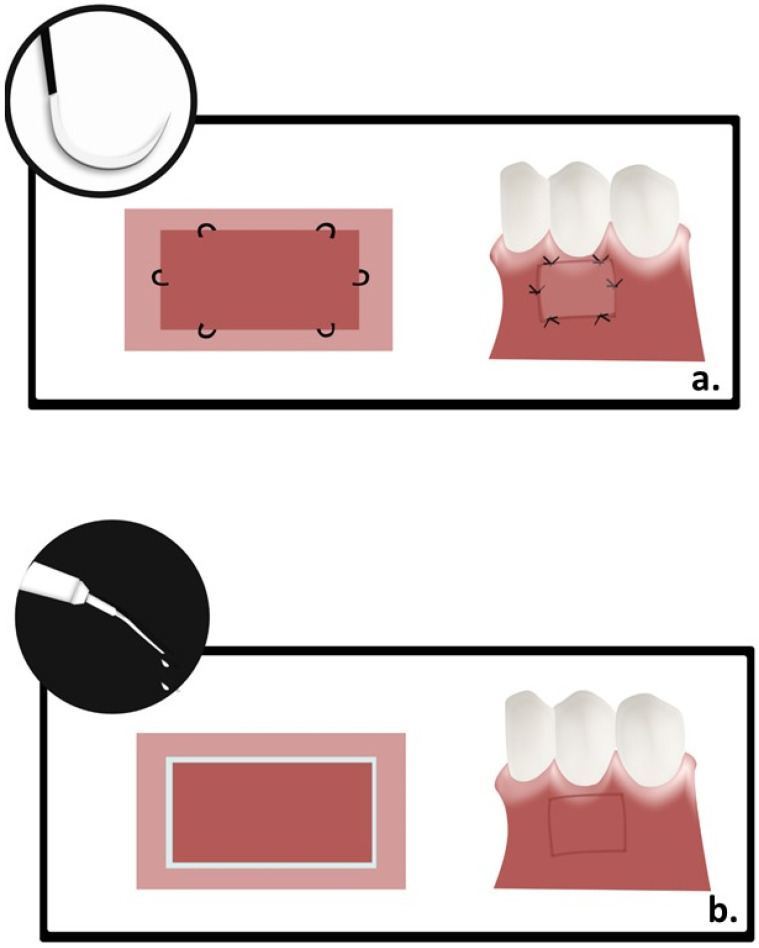
(**a**).—Free gingival graft stabilization in control group.; (**b**).—free gingival graft stabilization in test group.

In control group, sutures were used for FGG stabilization as explained previously [1]. Briefly, the graft was held in place using three simple interrupted sutures in coronal portion, and another three in apical portion using 5-0 propylene sutures (Ethicon, Johnson & Johnson, USA). After checking the graft stability by air blow method, gentle pressure was exerted onto the graft for five minutes, while, in test group, butyl cyanoacrylate (PeriAcryl^®^, GluStitch, Delta, Canada) was used to hold the FGG in place. After applying gentle pressure to FGG for five min, cyanoacrylate was applied using a 0.2 mL pipette to cover about 2 mm width of adhesive alongside the FGG borders. Excessive adhesive was wiped off using a gauze moistened with sterile saline. A set time of 1–2 min was allowed to ensure the adherence of FGG to the recipient site (Figure 2).

### 2.4. Post-Surgery Instructions and Follow-Up Visits

Participants were instructed to refrain toothbrushing or flossing at the surgical sites for the three postoperative days and advised to rinse twice a day using 0.12% chlorhexidine gluconate solution. Unless contraindicated, ibuprofen (600 mg) was prescribed to control postoperative pain and was instructed to use when needed only. In addition, participants were advised to apply ice packs during the first 48 h. During first week, participants were advised to consume only soft diet, prevent any mechanical trauma, and minimize lip movement while talking or smiling. Each participant was scheduled at the following post-operative visits: weekly for the first 4 weeks, then after 1, 2, 3 and 4 months, respectively. Upon each follow-up visit, professional plaque control was performed along with revisiting oral hygiene instructions. All clinical measurements were obtained in the same manner as the baseline by a single trained and calibrated (A.K.) which was also masked as to the intervention used in each site examined.

### 2.5. Assessment of Clinical Parameters

We utilized the gingival bleeding index (GBI) and full-mouth visible plaque index (VPI) to evaluate the oral hygiene and gingival health at baseline and throughout the study [15,16]. The following clinical parameters were measured on mid-buccal aspect of the included teeth in present study: clinical attachment level (CAL), determined from cemento-enamel junction to base of the sulcus; periodontal pocket depth(PD), measured from gingival margin (GM) to bottom of the gingival sulcus; apico-coronal width of keratinized tissue (KTW), measured from the muco-gingival junction(MGJ) to the GM; gingival tissue thickness (GTT), calculated 2 mm apically to GM. All measurements were obtained using William’s periodontal probe (Hu-Friedy, USA) at the nearest 1 mm. For the measurement of GTT, an endodontic finger spreader with a rubber stopper was inserted perpendicular to tooth, 2 mm apical to GM, until it contacted the root surface followed by moving the rubber stopper gently until it approached the soft tissue. Gingival thickness was then measured as the distance from the tip of the endodontic finger spreader tip to the rubber stopper [17,21] Following formulae were used to calculate the area and percent shrinkage of FGG area [22]:

Area = length × length

Shrinkage (%) = 100 × ([baseline dimension-postoperative dimension]/baseline dimension)

All measurement were performed at Baseline (BL), 15, 30, 60, 90 and 120 days postoperatively [18].

### 2.6. Post-Surgery Questionnaire during Follow-Up Visits

A preoperative questionnaire was filled out by all participants, which dealt with questions related to demographic and systemic health. Postoperative pain, days and number of analgesic tablets taken were also evaluated using questionnaires processed at the baseline to week 4 postoperative appointments. The questionnaires data evaluated the postoperative pain of participants using a visual analog scale (VAS) scores from ‘0’ indicating no pain at all to ‘10’ representing severe pain. In addition, participants were asked to locate the pain whether it was at the recipient site, donor site, or elsewhere in the oral cavity [23].

### 2.7. Data Analysis

The present study analyzed all data using SPSS software (v26.0, IBM Inc., Chicago, IL, USA). Descriptive analyses (mean, standard deviation, median and Inter Quartile range) were used to describe the symmetric and skewed quantitative outcome variables. One-way repeated measures analysis of variance followed by post hoc test were performed to compare mean values of outcome variables at 6 different time points of observation (baseline 15, 30, 60, 90 and 120 days). In each of the two study groups (test and control) a non-parametric repeated measures Friedman test was further utilized to compare mean ranks of FGG shrinkage% values at the 6 time points of observation in each of the two study groups (test and control). Student’s paired t-test was performed to compare mean values of KTW, GTT and VAS scores between test and control groups at each time point of observations. A non-parametric Wilcoxon signed ranks test was applied for comparing the mean differences of all parameters between the experimental and control groups at 15, 30, 60, 90 and 120 days of observation except for VAS, which was taken. The statistical significance was set at a *p*-value of ≤0.05.

## 3. Results

A total of 22 participants was involved in this study, where each side (from 2nd premolar to central incisor) was randomized to either control or test group. The outcome variables were KTW, GTT, VAS scores and FGG shrinkage%. All parameters were observed at six time points (baseline (BL) 15, 30, 60, 90 and 120 days) except for VAS, which was taken at three time points (BL, 15, 30), as pain was completely resolved afterwards from both study groups by the third appointment. The comparison of the mean values of KTW, GTT, VAS scores and mean ranks of FGG shrinkage% in the test group showed insignificant differences in the mean values of KTW across the six time points of observation (F = 1.0; *p* = 0.422), whereas there was a highly statistically significant difference in the mean values of GTT across the six time points of observation (F = 3.32; *p* = 0.008). The post hoc test indicated that the mean GTT values were significantly higher at BL and at 15 days when compared with the mean values of GTT values at 30, 60, 90 and 120 days, and no difference in the mean GTT values across these four points of observation (30, 60, 90 and 120 days) (Table 2).

Additionally, there was a statistically significant difference in the mean values of VAS scores across all time points of observation (F = 151.94, *p* < 0.0001), where the mean VAS score at BL was significantly higher and it had significantly reduced at 15 days and further significantly reduced at 30 days. Additionally, there was a highly statistically significant difference in the mean ranks of FGG shrinkage% values across the five time points of observations (15, 30, 60, 90 and 120 days), where the FGG shrinkage% values significantly increased from 15 days up to 120 days (*p* < 0.0001).

The post hoc test indicated that the mean GTT values were significantly higher at BL and at 15 days when compared with the mean values of GTT values at 30, 60, 90 and 120 days, and no difference in the mean GTT values across these four points of observation (30, 60, 90 and 120 days). Additionally, there was a highly significant difference in the mean values of VAS scores across the three time points of observation (F = 186.69, *p* < 0.0001), where the mean VAS score at BL was significantly higher and it had significantly reduced at 15 days and further significantly reduced at 30 days. Additionally, there were highly significant differences in the mean ranks of FGG shrinkage% values across the five time points of observations (15, 30, 60, 90 and 120 days), where the FGG shrinkage% values significantly increased from 15 days up to 120 days (*p* < 0.0001).

The comparison of the mean values of KTW, GTT and VAS scores between the test and control groups showed statistically insignificant differences in the mean values of KTW at each of the six time points of observations (BL, 15, 30, 60, 90 and 120 days). The mean KTW values were comparable in both groups at each of these six time point observations. On the other hand, there was a significant difference in the mean values of GTT between the test and control groups at each of the six time points of observations. That is, the mean GTT values of the test group were statistically significantly lower at BL, 15, 30, 60, 90 and 120 days when compared with the mean GTT values of the control group. However, there was a statistically insignificant difference in the VAS scores while comparing the experimental and control groups at BL, 15 and 30 days of observation. In addition, there was no significant difference in the mean ranks of FGG shrinkage% between test and control groups at 15, 30, 60, 90 and 120 days of observation (Table 3 and Table 4).

As for the number of postoperative pain days, the CAA groups showed significantly less pain days when compared to the suturing group (2(0.5) days vs. 6 (1.2) days, *p* < 0.0001). As for the number of analgesic tablets used, both groups reported that they stopped using analgesics by day 3 on average, having the most constant use by day 1 with no significant difference in the number of tablets used during this 3-day period between both groups (*p* > 0.05).

## 4. Discussion

The free gingival graft used to increase the range of keratinized mucosa, the donor tissue thickness and the graft stabilization in the recipient area are vital to protect local vessels against damage and dehydration, thereby decreasing the possibility of bleeding, tissue retraction and, consequently, the contamination of the surgical wound and postoperative pain. It is possible that techniques which promote surgical wound closure without the use of sutures, for example, tissue bio-adhesives, may have a hemostatic effect and still decrease or, furthermore, prevent tissue retraction and its negative consequences.

The FGG wound healing process can be improved through reaching a proper wound edge approximation and optimizing good wound isolation. Graft contamination with plaque/food debris and the excessive mobilization of tissues may occur post-operatively, resulting in the production of excessive granulation tissue, causing delayed epithelialization/healing at the graft site [24]. The combined effects of discussed factors may result in the failure of the procedure to yield the expected outcome and postoperative pain/discomfort. The chances of infection are decreased by vigilant attention to asepsis and the appropriate management of the tissues. The protection of the tissues and sepsis control remain the major concern during the healing period. Healing is improved by the immobilization of the healing area through either suturing or using tissue adhesives such as CAA [25].

The main advantages and indication of FGG procedure include the modification of the phenotype of the autogenous soft tissue. In addition, increasing the keratinized tissue width to facilitate the maintenance of proper oral hygiene minimizes inflammation around natural teeth and dental implants [26,27]. Furthermore, obtaining a thick phenotype through FGG would prevent any future gingival recession [21,28,29]. Therefore, it is crucial to promote this surgical procedure outcome to provide the best success outcomes and, by that, provide the advantages discussed.

A new protocol was proposed to improve the stability and vascularity along with reducing the tension and laceration of the graft material when placed in the recipient site, being the use of CAA. In the present study, all efforts were performed to compare this new method to the well-standardized method used to stabilize the graft through conventional suturing material. Success outcomes were evaluated to reflect both objective outcomes of the procedure (KTW, GTT and shrinkage%) and subjective outcomes (VAS scores).

Results of the present study showed that cyanoacrylate had comparable results to suturing in KTW across all observed time points. Similarly, the FGG shrinkage percentage did not differ between the groups, as well as the pain which was assessed by utilizing the VAS score which showed comparable pain resolving during the same period of time between the test and control groups. However, the thickness of the graft (GTT) was significantly different between the two groups. The suture group showed a higher mean of thickness than the cyanoacrylate at the 30-day time point and beyond. This finding could be attributed to the cyanoacrylate reacting with tissues, which led to an exaggerated sloughing of the superficial layer of the epithelium.

The present study’s findings were in line with the clinical findings of Giray et al. [30], Barnett et al. [31], Parmar et al. [32] and Quinn et al. [33], who reported that the closure of the wound with CAA was painless as compared to the conventional techniques. The time taken for the closure of the wound using the CAA was less in comparison to the most commonly used 3-0 silk sutures. Bruns et al. [34] also reported a quick and fast application time for the CAA procedures. These phenomena might be attributed to the anti-bacterial effect of the CAA as stated by earlier research studies [25,30]. The CAA exhibited excellent results in terms of hemostasis. This was observed in patients treated with CAA, as immediate clotting (hemostasis) was achieved for them compared to patients treated with the suturing technique. Howard et al. [35] reported similar properties of acrylates as he achieved good hemostasis during the healing of tooth extraction sites using bucrylates. In addition to these advantages, the CAA-treated patients were also found to have a lesser amount of post-operative inflammation as reported earlier by Kulkarni et al. [36] and Kumar et al. [37].

These findings corroborated what has been reported in the literature. In a split mouth study, Paknejad et al. (2004), in which they compared EPIGLU and silk sutures, it was found that both had a good stability with no significant difference between the two groups in pain perception or graft shrinkage [38]. Furthermore, a long-term study comparing free gingival grafts stabilized by CAA and sutures also showed no difference in graft shrinkage after six years. In addition, they showed similar outcomes to the present study in the tissue thickness, where the grafts in the cyanoacrylate group were shown to be thinner when evaluated at follow-up appointments [39].

The present study showed that cyanoacrylate, as an alternative method to stabilize a free gingival graft, is a worthwhile option that could help reduce the time and effort during the procedure.

Another similar material has been also highlighted as an alternative for conventional suturing in periodontal surgeries, named the Fibrin Fibronectin Sealing System (FFSS) [18]. It is available as a two component system: the first component contains highly concentrated fibrinogen, factor XIII, fibronectin and traces of other plasma proteins. The second component contains thrombin, calcium chloride and antifibrinolytic agents such as aprotinin. The mixing of the two components promotes clotting with the formation and cross-linking of fibrin [19]. It has been claimed to be effective in fixing tissues after periodontal surgery, as fibrin glue is easier and quicker to use than sutures. Sutures cause inflammation around themselves, while fibrin glue enhances early wound healing. In periodontal plastic surgeries of esthetically important areas, it gives better results than sutures. However, some limitations have been reported, which included a relatively higher cost than CAA, required some time as several vials needed be mixed and processed before applying it to the area, and it not being favorable to be used in areas with massive bleeding. The CAA material needs further investigation [18,19,20].

Limitations of the present study included a small sample size due to the difficulty to recruit participants with such strict criteria, but relatively short follow-up periods which were resistant to participants’ attrition. Additionally, the bleeding time and analgesic consumption were not assessed; it can be stated that the CAA seemed to be a promising material to be used in stabilizing FGG during and after the procedure, which could give comparable success outcomes to contention suturing. It can also be stated that CAA has several advantages to suturing due to easiness with a lower time consumption and being relatively cost effective, as it only takes a few minutes to apply a coat to secure the graft. However, it is important to be cautious about its reactivity and possible allergic reaction due its content of a polymer which, in case it occurs, can be controlled by the prescription of an oral anti-histamine. Overall, these observations need to be further investigated through larger scales of randomized clinical trials with a larger sample size and more standardized indices to assess healing outcomes with additional comparisons to various types of suturing materials and techniques.

## 5. Conclusions

The stabilization of free gingival grafts with cyanoacrylate is comparable to the conventional suturing of the grafts in terms of success outcomes. The use of cyanoacrylates decreased the operation time and post-operative inflammation. With the property of faster wound healing and cost effectiveness, cyanoacrylates can be considered as a favorable alternative for conventional and microsurgical approaches for the stabilization of free gingival grafts in the oral cavity.

## Figures and Tables

**Table 1:** Inclusion and exclusion criteria of the participating patients.

Inclusion Criteria	Exclusion Criteria
Adult of ages 18 years and above with healthy periodontium.	Patients who were smokers.
Patients who presented with bilateral insufficient keratinized tissue width (KTW) of less than 2 mm or thin phenotype in lower anterior.	Patients suffering from systemic diseases, that may impair the normal healing process.
Patients with teeth areas that were indicated for autogenous free gingival graft (FGG) pre-proclination movement during orthodontic treatment.	Patients undergoing radiotherapy of head and neck region.
Patients with normal level of crestal bone on these teeth radiographically.	Patients with bisphosphonate treatment.
Patients with absence of probing depth (PD) >3 mm.	Patients who refused to participate.
Patients with absence of supragingival and/subgingival calculus.	

**Table 2:** Comparison of mean values and mean ranks of outcome variables (KTW, GTT, VAS score and FGG shrinkage%) across the 6 time points of observation in a test group.

Outcome Variables	Time Points (in Days)						F-Value	*p*-Value
	BL	15	30	60	90	120		
**KTW**	**3.14 (1.04)**	3.14 (1.04)	3.14 (1.04)	3.14 (1.04)	3.09 (1.02)	3.09 (1.02)	1	0.422
**GTT**	2.14 (0.35)	2.14 (0.35)	2.0 (0)	2.0 (0)	2.0 (0)	2.0 (0)	3.32	0.008
**VAS score**	5.59 (1.59)	2.55 (1.01)	0.50 (0.80)	--	--	--	151.94	<0.0001
**FGG Shrinkage%** **Median (IQ)** **Mean Ranks**	--	0.0 (36)	12.5 (36)	20.8 (23.5)	31.5 (17.8)	36 (31.1)	--	--
	--	1.95	2.36	3.05	3.61	4.02	--	<0.0001

**Table 3:** Comparison of mean values and mean ranks of outcome variables (KTW, GTT, VAS score and FGG shrinkage%) across the 6 time points of observation in a control group.

Outcome Variables	Time Points (in Days)						F-Value	*p*-Value
	BL	15	30	60	90	120		
**KTW**	**3.14 (1.04)**	3.14 (1.04)	3.14 (1.04)	3.14 (1.04)	3.09 (1.02)	3.09 (1.02)	1	0.422
**GTT**	2.50 (0.51)	2.50 (0.51)	2.36 (0.49)	2.36 (0.49)	2.36 (0.49)	2.32 (0.48)	3.4	0.007
**VAS score**	5.64 (1.53)	2.55 (1.06)	0.45 (0.67)	--	--	--	186.69	<0.0001
**FGG Shrinkage%** **Median (IQ)** **Mean Ranks**	--	0.0 (28)	25 (20.5)	25 (15.5)	26.5 (6)	28 (16)	--	--
	--	1.91	2.61	2.82	3.59	4.07	--	<0.0001

**Table 4:** Comparison of mean values of outcome variables (KTW, GTT, VAS score and FGG shrinkage%) between control and test groups at each of the time point observations.

**Outcome Variables**	**Time Points (Days)**	**Group**		**t-Value**		***p*-Value**
		**Test**	**Control**			
**KTW**	**BL**	**3.14 (1.04)**	**3.14 (1.04)**	--		--
	15	3.14 (1.04)	3.14 (1.04)	--		--
	30	3.14 (1.04)	3.14 (1.04)	--		--
	60	3.14 (1.04)	3.14 (1.04)	--		--
	90	3.09 (1.02)	3.09 (1.02)	--		--
	120	3.09 (1.02)	3.09 (1.02)	--		--
**GTT**	BL	2.14 (0.35)	2.50 (0.51)	2.75		0.009
	15	2.14 (0.35)	2.50 (0.51)	2.75		0.009
	30	2.0 (0.0)	2.36 (0.49)	3.64		0.001
	60	2.0 (0.0)	2.36 (0.49)	3.64		0.001
	90	2.0 (0.0)	2.36 (0.49)	3.64		0.001
	120	2.0 (0.0)	2.32 (0.48)	3.13		0.001
**VAS score**	BL	5.59 (1.59)	5.64 (1.53)	0.097		0.924
	15	2.55 (1.01)	2.55 (1.06)	0		1
	30	0.50 (0.80)	0.45 (0.67)	−0.20		0.84
		**Median (IQR)**	**Median (IQR)**	**Mean Ranks**		***p*-Value**
				**Negative**	**Positive**	
**FGG Shrinkage%**	15	0.0 (36)	0.0 (28)	4	8.5	0.493
	30	12.5 (36)	25 (20.5)	5	12	0.14
	60	20.8 (23.5)	25 (12.5)	5.8	11	0.373
	90	30.5 (17.8)	26.5 (6)	5.67	9	0.342
	120	36 (31.1)	28 (16)	6.41	11.5	0.257

## Data Availability

Data is available on request from corresponding author.

## References

[1] Sullivan H.C., Atkins J.H. (1968). Free autogenous gingival grafts. I. Principles of successful grafting. Periodontics.

[2] Nettem S., Nettemu S.K., Singh V.P., Nayak S.U. (2018). Free gingival graft: An effective technique to create healthy keratinized gingiva. Indian J. Mednodent Allied Sci..

[3] Stavropoulou C., Atout R.N., Brownlee M., Schroth R.J., Dmd A.K. (2019). A randomized clinical trial of cyanoacrylate tissue adhesives in donor site of connective tissue grafts. J. Periodontol..

[4] Grisdale J. (1998). The use of cyanoacrylates in periodontal therapy. Can. Dent Assoc..

[5] Donoff R.B. (1976). Biological basis for vestibuloplasty procedures. J. Oral Surg..

[6] Harris R.J. (2001). Clinical evaluation of 3 techniques to augment keratinized tissue without root coverage. J. Periodontol..

[7] Holbrook T., Ochsenbein C. (1983). Complete coverage of the denuded root surface with a one-stage gingival graft. Int. J. Periodontics Restor. Dent..

[8] Miller P.D. (1988). Regenerative and reconstructive periodontal plastic surgery. Mucogingival surgery. Dent. Clin. N. Am..

[9] Levin M.P., Cutright D.E., Bhaskar S.N. (1975). Cyanoacrylate as a periodontal dressing. J. Oral Med..

[10] Brauer G.M., Jackson J.A., Termini D.J. (1979). Bonding of acrylic resins to dentin with 2-Cyanoacrylate esters. J. Dent. Res..

[11] Barkhordar R.A., Javid B., Abbasi J., Watanabe L.G. (1988). Cyanoacrylate as a retro filling material. Oral Surg. Oral Med. Oral Pathol. Oral Radiol. Endod..

[12] Singer A.J., Quinn J.V., Clark R.E., Hollander J. (2002). Closure of lacerations and incisions with octylcyanoacrylate: A multicenter randomized controlled trial. Surgery.

[13] Singer A.J., Quinn J.V., Hollander J.E. (2008). The cyanoacrylate topical skin adhesives. Am. J. Emerg. Med..

[14] Vickers A.J. (2006). How to randomize. J. Soc. Integr. Oncol..

[15] Lindhe J., Meyle J. (2008). Peri-implant diseases: Consensus Report of the Sixth European Workshop on Periodontology. J. Clin. Periodontol..

[16] Lang N.P., Adler R., Joss A., Nyman S. (1990). Absence of bleeding on probing An indicator of periodontal stability. J. Clin. Periodontol..

[17] Listgarten M.A. (1980). Periodontal probing: What does it mean?. J. Clin. Periodontol..

[18] Pini Prato G.P., Cortellini P., Clauser C. (1988). Fibrin and fibronectin sealing system in a guided tissue regeneration procedure. A case report. J. Periodontol..

[19] Jathal B., Trivedi A., Bhavsar N. (2008). Use of fibrin glue in periodontal flap surgery. J. Indian Soc. Periodontol..

[20] Pulikkotil S.J., Nath S. (2013). Fibrin sealant as an alternative for sutures in periodontal surgery. J. Coll. Physicians Surg. Pak..

[21] Barootchi S., Tavelli L., Zucchelli G., Giannobile M.V., Wang H.L. (2020). Gingival phenotype modification therapies on natural teeth: A network meta-analysis. J. Periodontol..

[22] Cifcibasi E., Karabey V., Koyuncuoğlu C., Düzağaç E., Genceli E., Kasali K., Çintan S. (2015). Clinical evaluation of free gingival graft shrinkage in horizontal and vertical dimensions. J. Istanb. Univ. Fac. Dent..

[23] Haefeli M., Elfering A. (2006). Pain assessment. Eur. Spine J. Off. Publ. Eur. Spine Soc. Eur. Spinal Deform. Soc. Eur. Sect. Cerv. Spine Res. Soc..

[24] Toriumi D.M., Raslan W.F., Friedman M., Tardy M.E. (1990). Histotoxicity of cyanoacrylate tissue adhesives: A comparative study. Arch. Otolaryngol.–Head Neck Surg..

[25] Vaaka P.D., Patlolla B., Donga S., Ganapathi A., Kurapati V. (2018). Cyanoacrylate: An alternative to silk sutures: A comparative clinical study. J. Dr. NTR Univ. Heal. Sci..

[26] Iorio-Siciliano V., Blasi A., Sammartino G., E Salvi G., Sculean A. (2020). Soft tissue stability related to mucosal recession at dental implants: A systematic review. Quintessence Int..

[27] Kim D.M., Bassir S.H., Nguyen T.T. (2020). Effect of gingival phenotype on the maintenance of periodontal health: An American Academy of Periodontology best evidence review. J. Periodontol..

[28] Scheyer E.T., Sanz M., Dibart S., Greenwell H., John V., Kim D.M., Langer L., Neiva R., Rasperini G. (2015). Periodontal Soft Tissue Non–Root Coverage Procedures: A Consensus Report From the AAP Regeneration Workshop. J. Periodontol..

[29] Fischer K.R., Kunzlberger A., Donos N., Fickl S., Friedmann A. (2018). Gingival biotype revised-novel classification and assessment tool. Clin. Oral Investig..

[30] Giray C.B., Atasevar A., Durgun B., Araz K. (1997). Clinical and electronic microscope comparison of silk sutures and n-butyl-2-cyanoacrylate in human mucosa. Aus. Dent. J..

[31] Barnett P., Jarman F.C., Goodge J., Silk G., Aickin R. (1998). Randomized trial of histoacryl blue tissue adhesive glue versus suturing in the repair of paediatric facial lacerations. J. Paediatr. Child Health.

[32] Parmar H.D., Bhatt S.D. (2012). The sutureless circumcision- an alternative to the standard technique. Natl. J. Med. Res..

[33] Quinn J.V., Drzewiecki A., Li M.M., Stiell I.G., Sutcliffe T., Elmslie T.J., Wood W.E. (1993). A randomized, controlled trail comparing tissue adhesive with suturing in the repair of paediatric facial lacerations. Ann. Emerg. Med..

[34] Bruns T.B., Simon H.K., McLario D.J., Sullivan K.M., Wood R.J., Anand K.J. (1996). Laceration repair using a tissue adhesive in a children’s emergency department. Pediatric.

[35] Howard D., Whitehurst V.E., Bingham R., Stanback J. (1973). The use of bucrylate to achieve hemostasis in tooth extraction sites. Oral Surg. Oral Med. Oral Pathol..

[36] Kulkarni S., Dodwad V., Chava V.K. (2007). Healing of periodontal flaps when closed with silk sutures and N-butyl cyanoacrylate: A clinical and histological study. Indian J. Dent. Res..

[37] Kumar M.S., Natta S., Shankar G., Reddy S.H.K., Visalakshi D., Seshiah G.V. (2013). Comparison between Silk Sutures and Cyanoacrylate Adhesive in Human Mucosa- A Clinical and Histological Study. J. Int. Oral Heal..

[38] Paknejad M., Soleymani Shayesteh Y., Esmaielieh A. (2004). Free gingival grafting; epiglu VS. silk thread suturing: A comparative study. J. Dent. Tehran Univ. Med. Sci..

[39] Jaeger U., Andreoni C., Kopp F.R., Strub J.R. (1987). Sutures vs. adhesives: Two fixation methods for free gingival grafts. A six-year follow-up study. Quintessence Int..

